# Lead and uranium sorptive removal from aqueous solution using magnetic and nonmagnetic fast pyrolysis rice husk biochars

**DOI:** 10.1039/c7ra13540h

**Published:** 2018-04-10

**Authors:** Shujuan Wang, Wei Guo, Fan Gao, Yunkai Wang, Yue Gao

**Affiliations:** School of Environmental Science and Engineering, North China Electric Power University Beinong Road 2 Beijing 102206 China gwfybj@ncepu.edu.cn +86 10 61773936 +86 10 61773936; Beijing Key Laboratory of New Technique in Agricultural Application, Beijing University of Agriculture Beinong Road 2 Beijing 102206 China; Analytical, Environmental and Geo-Chemistry, Vrije Universiteit Brussel Pleinlaan 2 Brussel 1050 Belgium

## Abstract

This paper discusses the sorption characteristics of Pb(ii) and U(vi) on magnetic and nonmagnetic rice husk biochars. The porosity, specific surface area, hydrophobility, and reusability of biochar were effectively improved (1–2 times) after magnetic modification. The optimum adsorption conditions were as follows: biochar loading was 0.4 g L^−1^, pH value was 7.0, and anion strength of NO_3_^−^ and PO_4_^3−^ were 0.01 mol L^−1^ for Pb(ii) and 0.04 mol L^−1^ for U(vi) respectively. Compared with U(vi), Pb(ii) had the faster adsorption rate and higher adsorption capacity on magnetic biochar (MBC). The adsorption experimental data were well fitted by pseudo-second-order kinetic and Langmuir isotherm models. The maximum adsorption capacity of Pb(ii) and U(vi) on MBC was 129 and 118 mg g^−1^ at 328 K respectively, which was significantly higher than that of other sources biochars. Pb(ii) was mainly bonded to biochar by physisorption but the adsorption of U(vi) on biochar was mostly chemisorption. Fe oxides in MBC noticeably improved the ion exchange and complexation action between biochar and metal ion especially for U(vi). The experimental results confirmed MBC material can be used as a cost-effective adsorbent for the removal of Pb(ii) and U(vi) and can be separated easily from aqueous solution when application.

## Abstract 2

This paper discusses the sorption characteristics of Pb(ii) and U(vi) on magnetic and nonmagnetic rice husk biochars.

## Graphical abstract

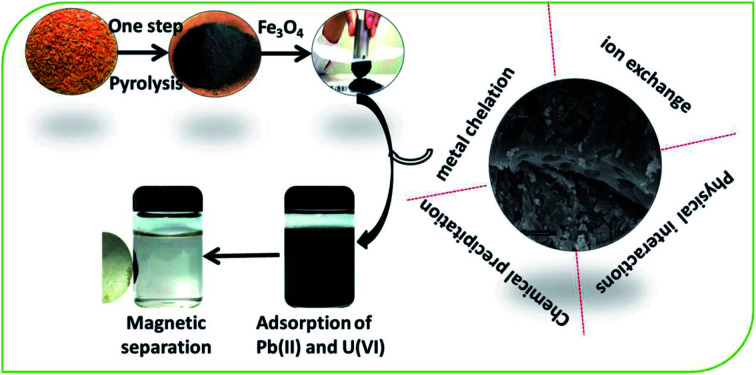

## Introduction

The rapid development experienced by the mining, metal smelting, electroplating, chemical engineering, and energy field industries in recent decades has led to serious environmental and health problems caused by heavy metals, especially in the industrialized areas of developing countries.^1^ China is the largest lead producer and consumer.^2^ Consequently, large amounts of wastewaters containing Pb(ii) were inevitably discharged into the aquatic environment of some places, causing negative effects such as elevated blood lead levels to numerous people.^3^ Moreover, China is a big country that is currently developing and using nuclear energy (130 580 GWh, 3% of the world nuclear power).^4^ Thus, nuclear radioactive elements such as U(vi) can be potentially discharged to the aquatic environment, and public health problems can be produced as a result of uranium mining and processing activities.

Pb(ii) and U(vi) are highly toxic heavy metals that can cause adverse damage on the ecosystem and human health at low concentrations.^5–8^ Therefore, considering the toxicity and the associated environmental and health risks of Pb(ii) and U(vi), along with their production and usage, the separation and removal of Pb(ii) and U(vi) from wastewaters have become urgent tasks in China and similar developing countries. According to the drinking water quality standard specified in the World Health Organization (WHO) guidelines, the maximum acceptable concentrations of Pb(ii) and U(vi) in drinking water should be lower than 10 and 15 μg L^−1^, respectively.^9,10^ With the aim to meet these stringent permissible Pb(ii) and U(vi) limits, water treatment utilities are required to upgrade their current technology or consider new purification techniques.

Different methods including precipitation, evaporation, ion-exchange, photocatalytic processes, reverse osmosis, coagulation–flocculation, ultrafiltration, and adsorption have been developed to treat Pb(ii) and U(vi) from the aqueous solution.^3,8^ Among these existing methods, adsorption has become one of the most promising the effective techniques due to its better performance, easy operation, and low cost. Thus, much effort has been spent developing inexpensive, environmental-friendly and higher effective adsorbents for the removal of toxic heavy metals.^11^ For example, Pb(ii) maximum adsorption capacities of 81.78 mg g^−1^ was determined for magnetic hydrogel beads,^12^ and the maximum sorption capacity of UO_2_^2+^ on poly(vinyl phosphonic acid) reached 900 mg g^−1^ at pH 6.^13^ Compared with these polymeric and commercial adsorbents, biochar, as a kind of biosorbents for heavy metals removal, has attracted much attention due to their special advantages of environmentally benign nature, low replacement cost, and practical application in a large scale.^14–16^ However, when directly applying these pristine biochars (BC) to wastewater treatment, the adsorption capacity and removal efficiency of pollutants on pristine biochar easily declined with the change of environmental factors such as pH, anion concentration, and temperature due to the structural and functional variation of biochar.^17,18^ Moreover, pristine biochar powders are difficult to separate from aqueous solutions after the removal treatment, thereby causing a secondary pollution problem.^19,20^ Previous studies have shown that a magnetic modification of biochar *via* aqueous Fe^2+^/Fe^3+^ solution or natural hematite treatments can effectively overcome these drawbacks.^21^ Thus, magnetic modification of biochars resulted in enhanced efficiencies towards the adsorption of some metals such as Cd(ii), Cr(iii), Pb(ii), and As(iii) while facilitating the recovery of the solid from the contaminated water *via* a filtration process with a magnet.^22–25^

Despite these novel studies, the adsorption mechanism and the behavior of magnetic biochar-adsorbed Pb(ii) and U(vi) in aqueous solutions under different influence factors (especially coexisting anions such as PO_4_^3−^ and NO_3_^−^) is not fully known. Additionally, studies on the adsorption characteristics of U(vi) on magnetic biochars (MBC) are particularly scarce in the literature. Considering this lack of knowledge, in the study, MBC were prepared from rice husk biochar, and the sorption efficiencies of Pb(ii) and U(vi) on MBC were compared with that on BC under different experimental conditions using batch experiments, and the adsorption mechanisms of Pb(ii) and U(vi) on BC and MBC were explored based on the analysis of biochar characterization. Finally, in view of a practical and sustainable application, the regeneration properties and stability of MBC were also investigated and evaluated. The study would offer a new alternative to transform biomass waste into a selective adsorbent for Pb(ii) and U(vi) removal from aqueous solution and also broad the applicability of biochar-based material in environmental pollution cleanup.

## Materials and methods

### Materials

A magnetically modified biochar was prepared *via* an aqueous Fe^3+^ solution treatment and subsequently was used to remove aqueous Pb(ii) and U(vi). Being inexpensive and abundantly available in China,^26^ rice husk was selected as a raw material to produce BC and MBC samples. Rice husk was obtained from the Carnival Farm Shop, Beijing, China. Stock solutions of Pb(ii) and U(vi) (200 mg L^−1^) were prepared by dissolving 0.320 g of Pb(NO_3_)_2_ and 0.333 g of H_2_N_2_O_8_U in 1 L of ultrapure water, respectively. Subsequently, these stock solutions were further diluted to the required concentration (40 mg L^−1^) with ultrapure water before performing the adsorption experiments. The pH of the experimental solutions was adjusted by using 0.1 M NaOH or HCl solutions. NaNO_3_ and Na_3_PO_4_ stock solutions (1 mol L^−1^) were prepared by dissolving the corresponding solid chemicals in ultrapure water.

### Preparation of the magnetic biochar

The rice husk was pyrolyzed to produce biochar at 773 K for 2 h in a tube furnace (SK-2.5-13, Beijing ZhongXing WeiYe Instrument Co., China) under a nitrogen flow rate of 10 mL min^−1^. The biochar was subsequently modified using hydrothermally synthesized magnetic iron oxide particles. As previously described by Trakal *et al.*,^23^ 1 g of FeSO_4_·7H_2_O was dissolved in 100 mL of water in a 500 mL beaker and a solution of NaOH (1 mol L^−1^) was slowly added under mixing until the pH value reached *ca.* 12.0. The suspension was subsequently water diluted to 200 mL and maintained at constant temperature (353 K, water bath) for 2 h. The beaker was subsequently removed from the water bath and the as-formed magnetic iron oxide particles were washed with water until the magnetic suspension reached neutral pH. The as-obtained iron oxide was Fe_3_O_4_. With the aim to produce magnetic biochar, 1 g of biochar powder was thoroughly mixed in a small beaker with 2 mL of the magnetic suspension at constant temperature (353 K, water bath) for 6 h. This mixture was dried completely at 333 K for 24 h. In the process of mixing, the Fe_3_O_4_ was subsided on the surface of biochar or into the pore structure of biochar.^24^ Finally, the dried material was reground to obtain small particles. Furthermore, the experiment of iron leaching from MBC within pH 2 to 11 was carried out to check the stability of MBC.^18^

### Biochar characterization

The as-prepared BC and MBC samples were analyzed as follows: (1) the specific surface area was determined on a Brunauer–Emmett–Teller surface area (*S*_BET_) analyzer (ASAP 2020, Micromeritics, USA); (2) the surface morphology and structure were examined by scanning electron microscopy (SEM, S250MK3, Cambridge UK Co., UK); (3) the high resolution structure of the materials were determined by transmission electron microscopy (TEM, JEM-2010, Japanese electronic optical CO., LTD); (4) the elemental analysis was carried out on an elemental analyzer (Vario EL, German Elementar Co., Germany); (5) the functional groups on the biochars before and after metal loading were determined by Fourier transform infrared spectroscopy (FTIR, Germany BRUKER spectrometer Co., Germany); (6) the phase composition was analyzed by X-ray diffraction (XRD, Empyrean, Netherlands); (7) the pH_zpc_ was measured on a ZETASIZER 3000 HSA system (Zetasizer Nano, UN); (8) the binding energy of the Pb(ii)- and U(vi)-loaded biochars on the surface and *ca.* 10 nm in deep were determined by X-ray photoelectron spectroscopy (XPS, Omicron Nanotechnology, Ltd.) and the Casa XPS program was used for the analysis of the spectra; and (9) the thermal stability of biochars was verified by thermogravimetric analysis (TGA, Discovery TGA 5500, TA instruments CO., USA).

### Batch experiments

Batch mode adsorption studies were conducted to investigate the influence of some parameters used herein such as the biochar dosage (0–1.0 g L^−1^), the pH (2–11), the coexisting anion (NO_3_^−^ and PO_4_^3−^, 0.001–0.1 mol L^−1^), the contact time (10 min–24 h), the initial concentration (10–80 mg L^−1^) of Pb(ii) and U(vi), the reaction system temperature (298, 313, and 328 K), and the adsorption–desorption recycling time (1–5) during Pb(ii) and U(vi) adsorption. All the batch experiments were performed with 10 mL polyethylene (PE) tubes. The mixed solutions of all batch experiments were shaken at 150 rpm and 25 °C for 8 h. A specific method was used for the adsorption–desorption recycling experiments. After adsorption of Pb(ii) and U(vi), the BC and MBC samples were soaked in 0.01 M HCl, shaken for 24 h, and subsequently washed with water and alcohol. Finally, the as-obtained BC and MBC were dried at 348 K for further use.^27^ The suspension was filtered with a 0.45 μm polysulfone filter membrane. The concentration of Pb(ii) and U(vi) remaining in the supernatant solution was measured by inductively coupled plasma coupled with mass spectrometry (ICP-MS, Agilent 7500, USA). Each batch experiment involved three parallel samples, and a blank experiment was conducted following the same test procedure. The relative data error was generally within 5%. The removal efficiency and the adsorbed amount of Pb(ii) and U(vi) at equilibrium (*q*_e_ (mg g^−1^)) were calculated using eqn (1) and (2), respectively:^28^1
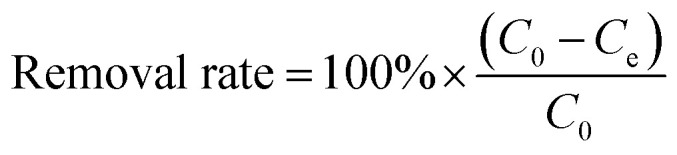
2
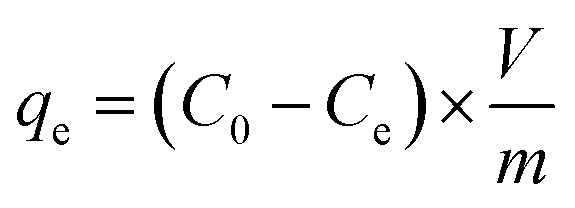
where *C*_0_ is the initial concentration of Pb(ii) or U(vi) (mg L^−1^), *C*_e_ represents the equilibrium concentration of Pb(ii) or U(vi) (mg L^−1^), *V* is the volume of the suspension (mL), and *m* is the weight of BC and MBC (g).

With the aim to understand the mechanism governing the adsorption of Pb(ii) and U(vi) over BC and MBC, pseudo-first-order and pseudo-second-order kinetic models were used to describe the adsorption kinetic data of Pb(ii) and U(vi). The pseudo-first-order and pseudo-second-order models can be described by the following equations:^2^

3 *q*_*t*_ = *q*_e_[1 − exp(−*K*_1_*t*)] (Pseudo-first-order)

4
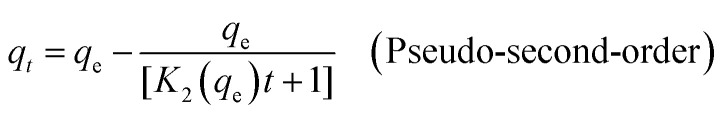
where *K*_1_ is the rate constant of the pseudo-first-order adsorption model (min^−1^), *K*_2_ is the rate constant of the pseudo-second-order adsorption model (g mg^−1^ min^−1^), and *q*_*t*_ and *q*_e_ (mg g^−1^) are the amounts of metal ions adsorbed at a contact time *t* (h) and at equilibrium, respectively.

The Langmuir and Freundlich models were applied to simulate the adsorption isotherms.^29^ The equations for the Langmuir and Freundlich models are described by the following equations:5
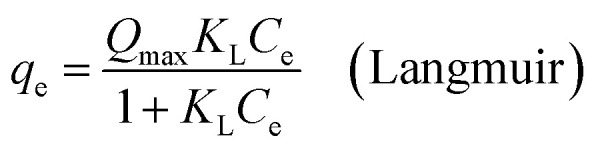

6 *q*_e_ = *K*_F_*C*_e_^1/*n*_F_^ (Freundlich)

where *q*_e_ is the equilibrium adsorption capacity of Pb(ii) and U(vi) on BC and MBC (mg g^−1^), *Q*_max_ is the maximum adsorption capacity (mg g^−1^), *K*_L_ is the Langmuir adsorption characteristic constant (L mg^−1^) related to the enthalpy of the adsorption process, *C*_e_ is the adsorption equilibrium concentration of Pb(ii) and U(vi) after equilibrium (mg L^−1^), *K*_F_ is the Freundlich equilibrium constant (mg g^−1^ (mg L^−1^)^−1/*n*_F_^), and *n*_F_ is dimensionless exponent of the Freundlich equation which varies with the degree of heterogeneity of adsorbing sites.

Moreover, thermodynamic parameters such as the Gibbs free energy (Δ*G*^0^), enthalpy (Δ*H*^0^), and entropy (Δ*S*^0^) changes were used to evaluate the feasibility and nature of the adsorption reaction. They were calculated using the following equations:

7 Δ*G*^0^ = −*RT* ln *K*

8 Δ*G*^0^ = Δ*H*^0^ − *T*Δ*S*^0^

9
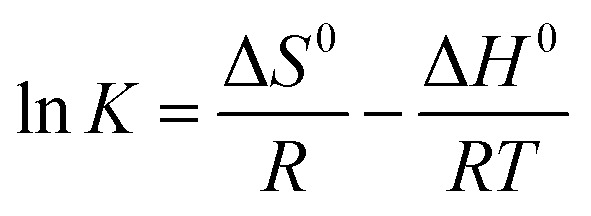
where *R* is the gas constant (8.314 J mol^−1^ K^−1^), *T* is the absolute temperature (K), and *K* is an equilibrium constant obtained by multiplying the Langmuir constants *Q*_max_ and *K*_L_.

## Results and discussion

### Characteristics of the biochars

Some selected physicochemical properties of BC and MBC are listed in Table 1. MBC (109 m^2^ g^−1^ and 0.05 cm^3^ g^−1^) showed higher BET surface area and micropore volume as compared to BC (52.1 m^2^ g^−1^ and 0.02 cm^3^ g^−1^), probably as a result of the addition of Fe_*x*_O_*y*_, in line with the results reported by Mohan.^30^ BC and MBC were both weakly alkaline (average pH values of 9.08 and 8.55, respectively). BC and MBC both showed low pH_pzc_ values (<5.0), thereby revealing high acidic characteristics and a strong buffer capacity under basic environments. The higher pH_pzc_ value of BC (4.32) might be ascribed to the presence of a higher amount of basic functional groups in this biochar.^31^ Moreover, the magnetite surface coverage is likely responsible for the lower pH_pzc_ of MBC (3.71).^32^ Iron ions were successfully adhered to BC, and the content in MBC was 8.49%. The oxygen content and the O/C ratio of BC were significantly higher than those of MBC. Thus, the former material was more hydrophilic because of the presence of a higher amount of basic functional groups.^30^

**Table 1:** Chemical and physical properties of the biochar samples^a^

Characteristics units	Biochars	
	BC	MBC
BET surface area, m^2^ g^−1^	52.1	109
*V*_p_, cm^3^ g^−1^	0.02	0.05
pH	9.08	8.55
pH_pzc_	4.32	3.71
C, %	56.3	40.7
H, %	2.05	1.65
O, %	35.9	21.4
N, %	0.71	0.47
S, %	0.16	0.12
Fe, %	—	8.49
O/C ratio	0.48	0.39
H/C ratio	0.44	0.49

a BC: biochar; MBC: magnetic biochar; *S*_BET_: surface area; *V*_p_: micropore volume.

**Fig. 1:**
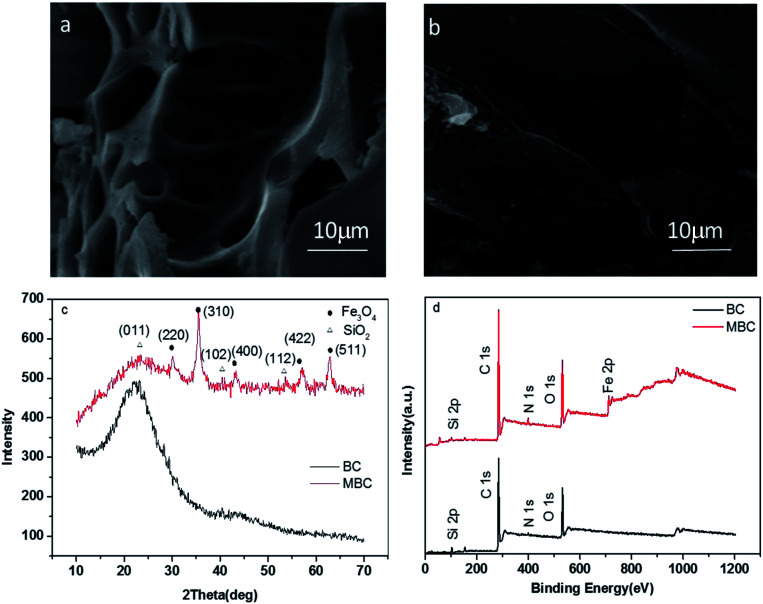
SEM images (a and b), XRD patterns (c), and XPS spectra (d) of BC and MBC, respectively.

**Fig. 2:**
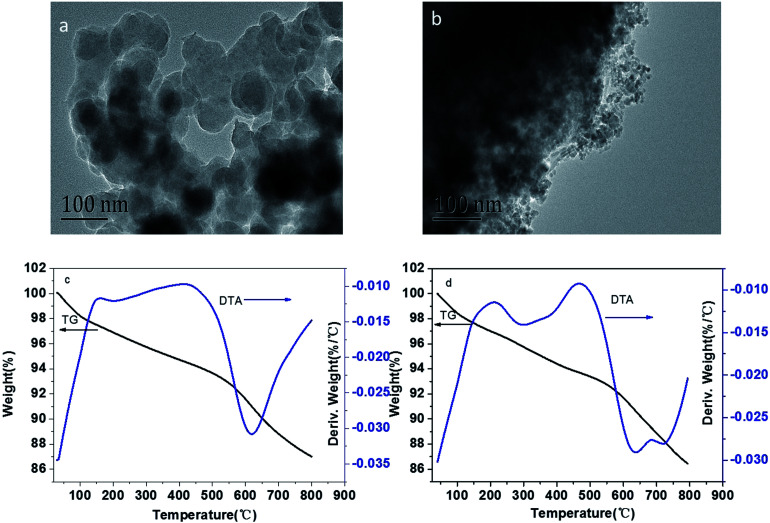
TEM images and TGA curves of BC (a and c) and MBC (b and d), respectively.

The structure and chemical composition of BC and MBC are shown in Fig. 1. As shown in the SEM micrographs (Fig. 1a and b), the magnetic modification process resulted in a biochar having a rough surface and abundant of micro-spheres and mineral particles, which can provide abundant adsorption points, further enhancing the adsorption capacity of the biochar.^24^ According to the XRD analysis, these micro-spheres and mineral particles in MBC might be formed by Fe_3_O_4_ (Fig. 1c). The five intense characteristic peaks detected at 2 theta = 30.5, 33.0, 41.7, 52.6, and 57.1° corresponded to the primary diffraction of the (220), (310), (400), (422), and (511) facets of a cubic spinel crystal Fe_3_O_4_ phase (JCPDS no. 19-692).^25^ To further analyze the surface chemical composition of the biochar samples, the XPS spectra (full scan 0–1200 eV) of BC and MBC were determined (Fig. 1d). The photoelectron lines at *ca.* 284, 400, and 533 eV corresponded to the C 1 s, N 1 s, and O 1 s signals of BC and MBC. Lines at binding energies of 710–721 eV corresponding to Fe 2p_3/2_ were detected for MBC and assigned to Fe^3+^ species, in line with the results reported by Trakal *et al.*^23^ The TEM images (Fig. 2a and b) of BC and MBC shown that a number of black spots were visible in Fig. 2b, which confirmed agglomerated, irregular, non-uniform, and spherical Fe_3_O_4_ particles were successfully adhered to BC *via* the hydrothermal synthesis method.^33^ Both BC and MBC showed no difference in their thermal stability (Fig. 2c and d), and they all experienced a three-step weight loss in the TG curve.^34^ During the first stage, a ∼2.5% weight loss occurred before 150 °C, which was due to the dehydration of physically adsorbed water molecules. Then a ∼7% weight loss occurred between 150 and 600 °C which was related with decomposition of hemicellulose, cellulose and lignin. A third stage, a 4–5% weight loss occurred within 600 and 800 °C, was probably due to the breakage of CO_3_^2−^.

### Effect of the biochar loading

**Fig. 3:**
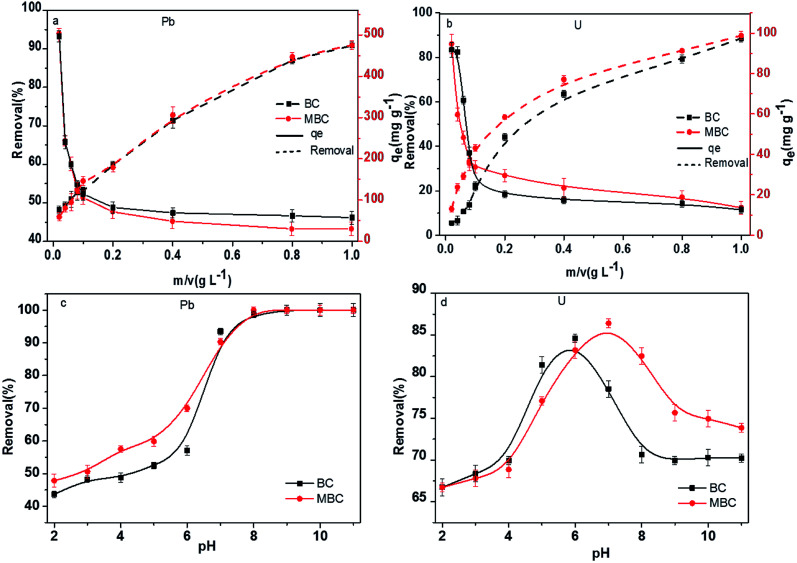
Removal of Pb(ii) (a) and U(vi) (b) in solution over BC and MBC as a function of the biochar dosage. Removal of Pb(ii) (c) and U(vi) (d) in solution over BC and MBC as a function of the pH.

The adsorbent dosage has been regarded as a crucial factor affecting the removal of contaminants.^35^ The influence of the biochar dosage on the Pb(ii) and U(vi) adsorption capacities is shown in Fig. 3. The Pb(ii) and U(vi) removal rates increased with the biochar dosage. As reported by Trakal *et al.*^23^ and Samsuri *et al.*,^36^ MBC showed higher removal rates than BC, especially for U(vi). Thus, while the removal rate of Pb(ii) in solution was similar for both biochar samples and increased linearly with the biochar loading (48.1 *vs.* 50.8% at 0.02 g L^−1^ and 90.9 *vs.* 91.7% at 1.00 g L^−1^ for BC and MBC, respectively, Fig. 3a), U(vi) in solution was more rapidly removed by MBC, and the removal rate increased nonlinearly with the biochar loading (5.5 *vs.* 13.0% at 0.02 g L^−1^ and 90.2 *vs.* 96.8% at 1.00 g L^−1^ for BC and MBC, respectively, Fig. 3b). While increasing the biochar loading is advantageous in that it provides more active sites for adsorption, excessive dosages can lead to adsorbent aggregation issues which reduce the number of binding sites and promote biochar–metal ions electrostatic repulsion.^37^ As shown in Fig. 3, BC and MBC both showed high Pb(ii) and U(vi) removal rates. From the economic and environmental viewpoints, 0.4 g L^−1^ was selected herein as the optimum biochar loading.

### Effect of the pH

The initial pH of the solution can significantly influence the adsorption of metals over biochars by altering the surface charge and the nature of the functional groups on the adsorbent, the metal ion speciation, or its solubility.^30^ Thus, the effect of the initial pH on the removal of Pb(ii) and U(vi) over BC and MBC was analyzed (Fig. 3c and d). The adsorption of Pb(ii) and U(vi) were noticeably affected by the pH of the solution. The removal efficiency of Pb(ii) showed a S-shaped curve with the pH, U(vi) showed an inverted U-shaped trend. At low pH values (2–5), Pb(ii) removal was inhibited because H^+^ competed with Pb(ii) for the adsorption sites. At low pH values, both BC (pH_zpc_ = 4.32) and MBC (pH_zpc_ = 3.71) showed positive charges, thereby hindering the contact between the Pb(ii) ions and the biochar surface *via* high electrostatic repelling forces. At higher pH values (pH > 5), some Pb(ii) hydrolysis products such as Pb(OH)_2_ were formed, and both adsorption and precipitation processes accounted for the removal of Pb(ii). However, at pH values lower than 8, Pb was mainly as Pb^2+^ in solution, and electrostatic attraction between Pb and biochar was the main adsorption driving force. At higher pH values (8–11), protonation–deprotonation processes of carboxyl and hydroxyl groups on the biochar prevailed,^38^ thereby inhibiting the adsorption of Pb(ii).

In the case of U(vi), the removal efficiency over BC and MBC first increased slowly with the pH (2–4), after which a rapid increase was observed (pH = 4–6 for BC and pH = 4–7 for MBC). At higher pH values (pH = 6–8 for BC and pH = 7–9 for MBC) the removal efficiency of U(vi) dropped significantly, and slowly decreased for pH values exceeding 8 (BC) and 9 (MBC). The low adsorption efficiency of U(vi) at low pH values (2–4) could be attributed to the competition of H^+^ with U(vi) for the adsorptive active sites and the higher positive charge of the biochar. At pH < pH_pzc_, the surface charge of the both biochars are positive because of the protonation reaction. Thus, the positive U(vi) ions are adsorbed with difficulty on the positively charged surface of the BC and MBC because of the electrostatic repulsion.^39^ At pH values above 6 (BC) and 7 (MBC), hydroxide U(vi) species such as UO_2_(OH)^+^, (UO_2_)_2_(OH)_2_, and (UO_2_)_3_(OH)_5_^2+^ were formed, thereby decreasing the removal ability of the biochar.^39^ The presence of Fe^3+^ on MBC might alter the interaction of OH^−^ with U(vi), thereby hindering the precipitation of hydroxides and reaching maximum removal rates at higher pH values (7 *vs.* 6) as compared to BC. BC and MBC both showed high removal rates for Pb(ii) and U(vi) at a pH of 7. Thus, this value was used for further Pb(ii) and U(vi) adsorption experiments on BC and MBC.

### Effect of the coexisting anions

**Fig. 4:**
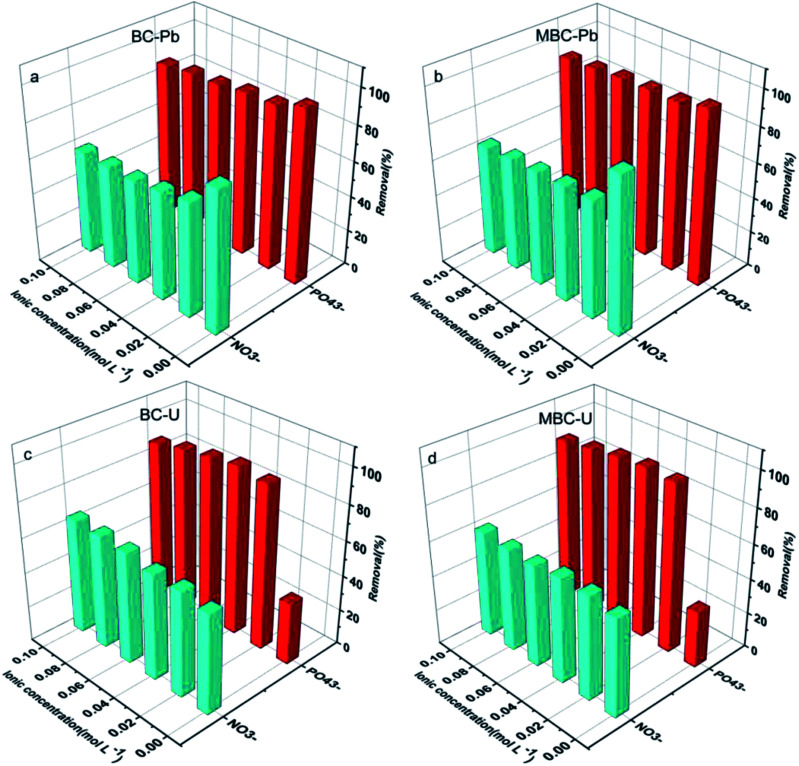
Effect of the ionic concentration of NO_3_^−^ and PO_4_^3−^ on the adsorption of Pb(ii) (a and b) and U(vi) (c and d) on BC and MBC, respectively.

Considering the complexity of water bodies (especially eutrophic waters), some coexisting anions such as NO_3_^−^ and PO_4_^3−^ might affect the uptake capacity of biochar towards the metal.^24^ The presence of NO_3_^−^ and PO_4_^3−^ significantly reduced the adsorption efficiency of Pb(ii) on BC and MBC, whereas the removal rate of U(vi) noticeably increased with the concentration of PO_4_^3−^ in solution for both biochar samples (Fig. 4). In the case of Pb(ii), the adsorption efficiency rapidly decreased with the concentration of NO_3_^−^ and PO_4_^3−^ increasing from 0 to 0.02 mol L^−1^ and moderately dropped at higher concentrations of coexisting anions on both biochars. While the adsorption efficiency of U(vi) slightly increased with the concentration of NO_3_^−^, a different trend was observed for PO_4_^3−^. Thus, the adsorption efficiency of U(vi) first increased rapidly with the concentration of PO_4_^3−^ (0–0.02 mol L^−1^) and moderately increased thereafter before reaching equilibrium. This phenomenon could be attributed to these reasons: (1) outer surface complexes were formed by interaction of Pb(ii) and biochar, with electrostatic attraction being the main adsorption mechanism of Pb(ii) over BC and MBC. As a result, the presence of NO_3_^−^ and PO_4_^3−^ in solution altered the electrostatic interaction between the surface charges of biochar and the Pb(ii) ions in solution while also competing with them for the surface adsorption sites. (2) Inner surface complexes were formed by interaction of U(vi) and biochar, with complex surface coordination and precipitation being the main mechanisms governing the adsorption of U(vi) over BC and MBC. Thus, the dissolution PO_4_^3−^ and UO_2_^2+^ contributed to form coordination and oversaturate conditions;^40^ (3) the ionic strength of NO_3_^−^ and PO_4_^3−^ influenced the activity coefficient of Pb(ii) to a higher extent as compared to U(vi), thereby limiting the transfer of Pb(ii) on the biochar surface. The optimum anion strengths of NO_3_^−^ and PO_4_^3−^ were 0.01 mol L^−1^ for Pb(ii) and 0.04 mol L^−1^ for U(vi), respectively.

### Adsorption kinetics

**Fig. 5:**
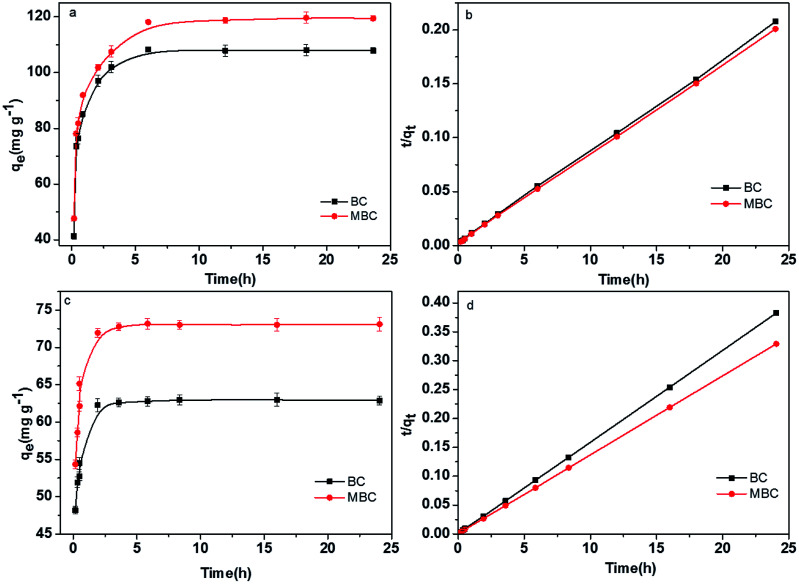
Effect of the contact time on the adsorption of Pb(ii) and U(vi) over BC and MBC. (a and c) Pseudo-first-order model for the adsorption of Pb(ii) and U(vi); (b and d) pseudo-second-order model for the adsorption of Pb(ii) and U(vi), respectively.

The effect of contact time on the adsorption of Pb(ii) and U(vi) on BC and MBC were depicted in Fig. 5. The pseudo-second-order model showed better *R*^2^ values than the pseudo-first-order model (Table 2). Thus, the pseudo-second-order model better described the adsorption of Pb(ii) and U(vi) on BC and MBC. This result revealed that, in addition to physical adsorption, chemical interactions were also involved in the adsorption of Pb(ii) and U(vi).^41,42^ Pb(ii) showed higher adsorption capacities and rates on BC and MBC as compared to U(vi). Additionally, the adsorption rate of Pb(ii) on BC was higher than that on MBC, thereby revealing that Pb(ii) was physically adsorbed on the biochar, while U(vi) was preferably adsorbed *via* chemisorption processes on MBC owing to the higher number of functional groups of this material.^40^

**Table 2:** Adsorption kinetics parameters for the adsorption of Pb(ii) and U(vi) on BC and MBC

Metals	Biochars	Pseudo-first order model			Pseudo-second order model		
		*q*_e_ (mg g^−1^)	*K*_1_ (min^−1^)	*R* ^2^	*q*_e_ (mg L^−1^)	*K*_2_ (g mg^−1^ min^−1^)	*R* ^2^
Pb(ii)	BC	104	0.41	0.938	126	0.05	0.946
	MBC	112	0.19	0.898	137	0.04	0.902
U(vi)	BC	61.0	0.27	0.938	90.1	0.07	0.999
	MBC	71.3	0.16	0.967	77.7	0.05	0.999

### Adsorption isotherms and thermodynamic characteristics

**Fig. 6:**
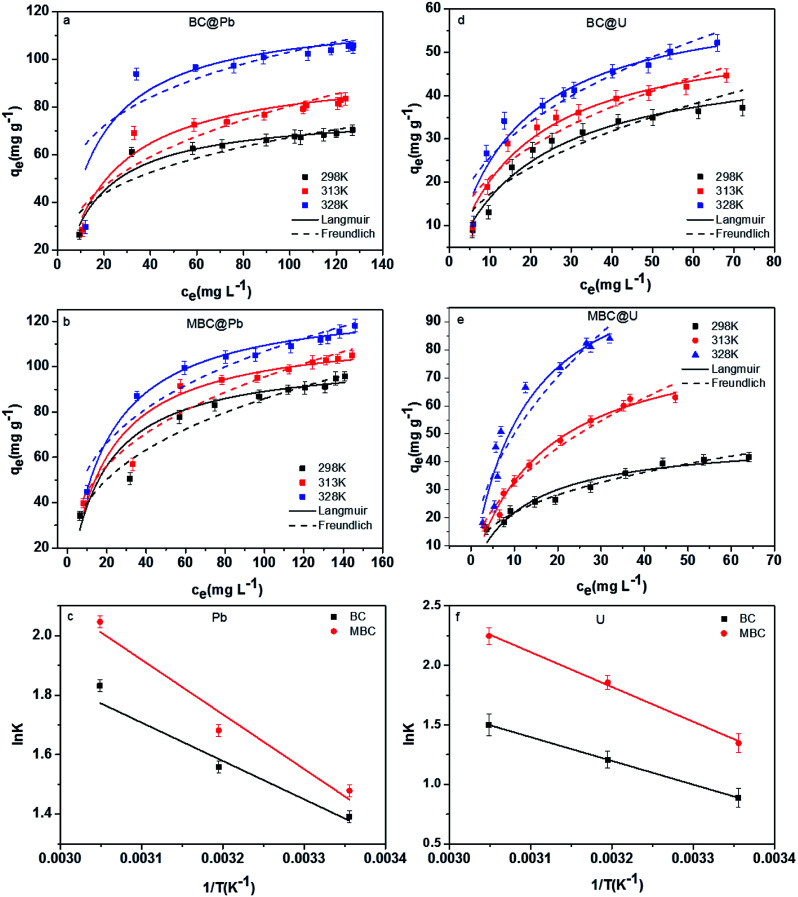
Adsorption isotherms and linear plot of ln *K vs.* 1/*T* for Pb(ii) (a–c) and for U(vi) (d–f) on BC and MBC, respectively.

Fig. 6 shows the adsorption isotherms and the thermodynamic characteristics of the adsorption of Pb(ii) and U(vi) on BC and MBC under three different temperatures (*i.e.*, 298, 313, and 328 K). The corresponding parameters for the Langmuir and Freundlich models are listed in Table 3. The coefficient correlation (*R*^2^) revealed that the Langmuir model provided a better fit to the adsorption of Pb(ii) and U(vi) on BC and MBC as compared to the Freundlich model (*R*^2^ = 0.87–0.99 *vs.* 0.73–0.96). This result further confirmed that Pb(ii) and U(vi) were adsorbed on the biochars forming a monolayer.^27^ The maximum adsorption capacities of Pb(ii) and U(vi) on BC and MBC dictated by the Langmuir model at 298 K were 77.5 and 110 mg g^−1^ for Pb(ii) and 48.6 and 53.2 mg g^−1^ for U(vi), respectively. The adsorption capacity of Pb(ii) had a more increase than that of U(vi) after biochar magnetic modification. The Pb(ii) removal potential from aqueous solutions by BC (77.5 mg g^−1^) and MBC (110 mg g^−1^) was significantly higher than other modified biochars (Table 4), such as those of oak wood-(10.1 mg g^−1^),^43^ oak bark-(30.2 mg g^−1^),^30^ bamboo-(25.1 mg g^−1^),^44^ pine bark-(25.3 mg g^−1^),^45^ romchar-(17.7 mg g^−1^) and oxford (32.2 mg g^−1^) derived biochars,^46^ and close to those of sewage-(99.8 mg g^−1^) derived biochars.^47^ For U(vi), the removal potential from aqueous solutions of the biochars tested herein (BC: 48.6 mg g^−1^, MBC: 53.2 mg g^−1^) was significantly higher than those of eucalyptus wood-(27.2 mg g^−1^),^48^ fungus pleurotus ostreatus- (19.4 mg g^−1^)^49^ and magnetic chitosan- (42 mg g^−1^)^51^ derived bichars. However, BC and MBC showed the lower U(vi) removal potential as compared to activated cactus fibre (214 mg g^−1^).^51^

**Table 3:** Parameters for the adsorption isotherm models and values of some thermodynamic parameters for the adsorption of Pb(ii) and U(vi) on BC and MBC

Metals	Biochars	*T*	Langmuir model			Freundlich model			Thermodynamic parameters		
			*Q*_max_ (mg g^−1^)	*K*_L_ (L mg^−1^)	*R* ^2^	*K*_F_	*n*	*R* ^2^	Δ*G*^0^ (kJ mol^−1^)	Δ*S*^0^ (J mol^−1^)	Δ*H*^0^ (kJ mol^−1^)
Pb(ii)	BC	298 K	77.5	0.07	0.941	17.3	0.26	0.813	−4.19	51.3	11.9
		313 K	95.0	0.05	0.943	19.8	0.30	0.842	−4.05		
		328 K	125	0.05	0.867	24.6	0.31	0.726	−5.00		
	MBC	298 K	110	0.04	0.967	18.9	0.33	0.958	−3.66	63.3	15.3
		313 K	122	0.04	0.972	22.4	0.32	0.958	−4.12		
		328 K	129	0.06	0.990	32.8	0.26	0.951	−5.58		
U(vi)	BC	298 K	48.6	0.05	0.961	7.64	0.39	0.953	−2.20	63.0	16.6
		313 K	55.8	0.06	0.969	9.47	0.38	0.953	−3.14		
		328 K	64.0	0.07	0.930	11.6	0.37	0.903	−4.09		
	MBC	298 K	53.2	0.08	0.931	9.58	0.36	0.981	−3.34	93.3	24.4
		313 K	87.4	0.06	0.984	10.7	0.48	0.970	−4.31		
		328 K	118	0.08	0.929	16.1	0.49	0.893	−6.13		

**Table 4:** Comparison of the maximum uptake of Pb(ii) and U(vi) on various modified biochar adsorbents

Adsorbate	Feedstock	Pyrolysis temperature (K)	Modified materials	BET surface area, (m^2^ g^−1^)	*Q*_max_ a (mg g^−1^)	Reference
Pb(ii)	Oak wood	673	Fe^2+^/Fe^3+^plus SO_4_^2−^ solution	2.04	10.1	Mohan *et al.* (2014)^43^
	Oak bark	673	Fe^2+^/Fe^3+^plus SO_4_^2−^ solution	25.4	30.2	Mohan *et al.* (2015)^30^
	Bamboo	—	Fine sized ZVI	—	25.1	Zhou *et al.* (2014)^44^
	Pine bark	1223	Ferrite(CoFe_2_O_4_)	—	25.3	Harikishore *et al.* (2014)^45^
	Romchar	773	Magnetite/maghemite	219	17.7	Han *et al.* (2015)^46^
	Oxford biochar	773	Magnetite/maghemite	68	32.2	
	Sewage sludge	673	Fe^2+^/Fe^3+^ plus SO_4_^2−^ solution without ZnCl_2_	103	99.8	Ifthikar *et al.* (2017)^47^
	Rice husk	773	Fe^2+^/Fe^3+^plus SO_4_^2−^ solution	109	110	In this study
U(vi)	Eucalyptus wood	673	—	20	27.2	Mishra *et al.* (2017)^48^
	Fungus pleurotus ostreatus	343	—	—	19.4	Zhao *et al.* (2016)^49^
	Magnetic chitosan	—	Magnetite powder	—	42	Stopa and Yamaura (2010)^50^
	Activated cactus fibre	—	—	<5	214	Hadjittofi and Pashalidis (2015)^51^
	Rice husk	773	Fe^2+^/Fe^3+^ plus SO_4_^2−^ solution	109	53.2	In this study

a *Q*_max_: the maximum Pb(ii) and U(vi) adsorption capacities of biochar in the water solution at 298 K. It was calculated based on the Langmuir model.

The values of Δ*H*^0^ and Δ*S*^0^ were obtained by plotting the lnK *vs.* 1/*T* (Fig. 6). Unlike Pb(ii) that showed similar maximum adsorption capacities at 328 K for both biochar samples (BC: 125 mg g^−1^ and MBC: 129 mg g^−1^), the adsorption of U(vi) was significantly enhanced over MBC (BC: 64.0 and MBC: 118 mg g^−1^). This could be explained by the energy of the adsorption system increasing with temperature (Table 3, ΔS^0^ and Δ*H*^0^ of MBC higher than those of BC, and the values of U(vi) higher than those of Pb(ii)), and the adsorption efficiency increasing as a result. The negative values of Δ*G*^0^ (from −6.13 to 2.20 KJ mol^−1^) suggested the adsorption of Pb(ii) and U(vi) on BC and MBC to be a spontaneous process being more favored at high temperatures.^1^ The positive value of Δ*S*^0^ revealed an increase in the randomness of the solid/solution interface.^52^ The positive value of Δ*H*^0^ revealed the adsorption of Pb(ii) and U(vi) on BC and MBC to be endothermic processes, while the low Δ*H*^0^ value could be indicative of a prevalent physical adsorption. High Δ*H*^0^ values are indicative of highly energetic processes involving the collapse of the hydration sheath of metal ions with molecular water and the formation of some chemical bonds between the metal ions.^53^

### Recycling of the biochars

**Fig. 7:**
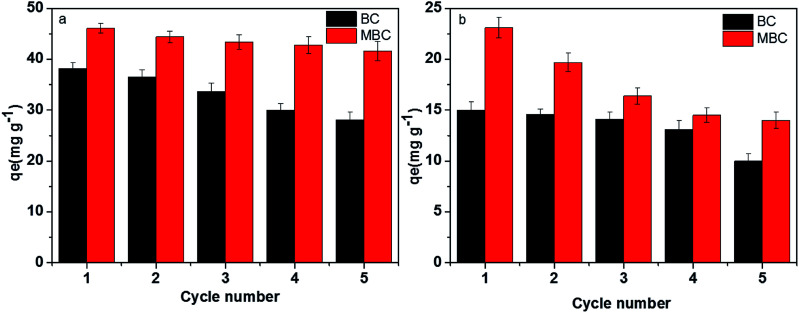
Recycling studies of BC (a) and MBC (b) for the adsorption of Pb(ii) and U(vi).

**Fig. 8:**
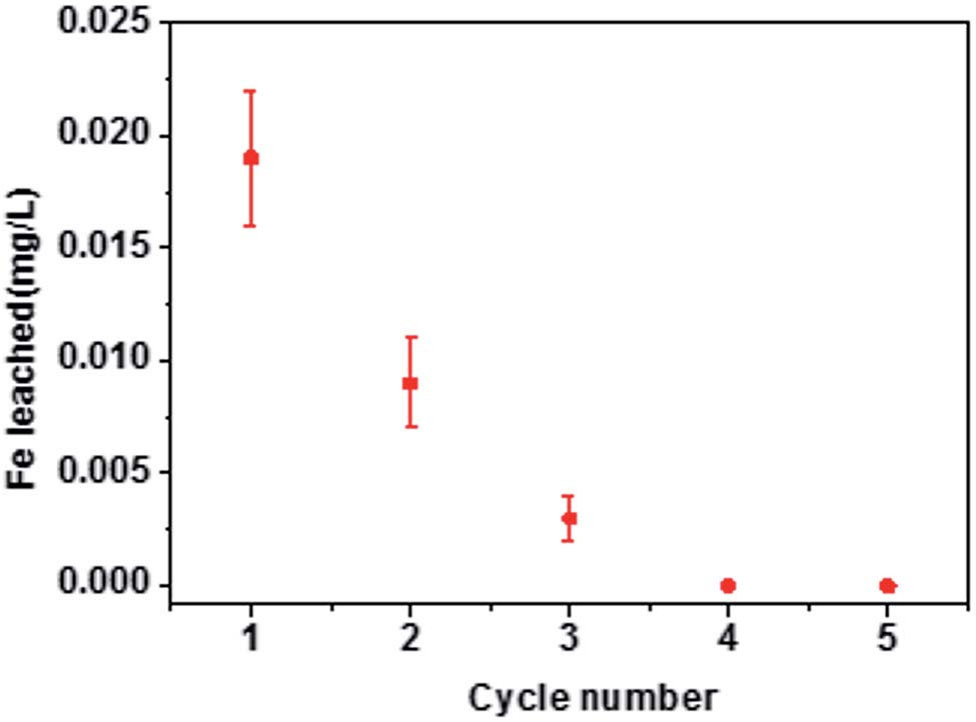
Stability of iron particles aggregated on MBC.

The economic value of BC and MBC was evaluated by performing a recycling study involving repetitive adsorption–desorption cycles. As shown in Fig. 7, the adsorption capacities of Pb(ii) and U(vi) on BC decreased from 38.1 to 25.3 mg g^−1^ and from 15.8 to 9.76 mg g^−1^, respectively, after five consecutive adsorption–desorption cycles. In contrast, the adsorption capacity of Pb(ii) on MBC only slightly decreased (from 47.3 to 40.8 mg g^−1^) after five consecutive adsorption–desorption cycles, whereas that of U(vi) significantly dropped from 28.1 to 13.7 mg g^−1^. Despite the decrease in the adsorption capacities of Pb(ii) and U(vi) on BC and MBC, the magnetically modified biochar showed a relatively high adsorption ability towards these metals and a good reusability behavior for Pb(ii) and U(vi) removal. Moreover, the amount of iron ions leaching from MBC in the solution was only 0.003–0.014 mg L^−1^ from the first cycle to the third cycle, and almost no iron ions was leached from MBC at the fourth cycle and the fifth cycle, which indicated the loaded iron on biochar was real stable.^18^ The observed decrease in the adsorption capacity could be explained in terms of: (1) a reduction in the surface area and pore volume of the biochar samples; (2) the presence of weaker functional groups; and 3) leaching of Fe_3_O_4_ on the surficial layer of biochar during the soaking process with a HCl solution(Fig. 8).^24,54^ The larger decrease in the adsorption efficiency of U(vi) on MBC as compared to Pb(ii) further confirmed the different adsorption behavior of both ions (*i.e.*, physisorption for Pb(ii) and chemisorption for U(vi)). Thus, the chemisorption process was more affected by the HCl soaking regeneration treatment of MBC as compared to physisorption.

### Adsorption mechanism

**Fig. 9:**
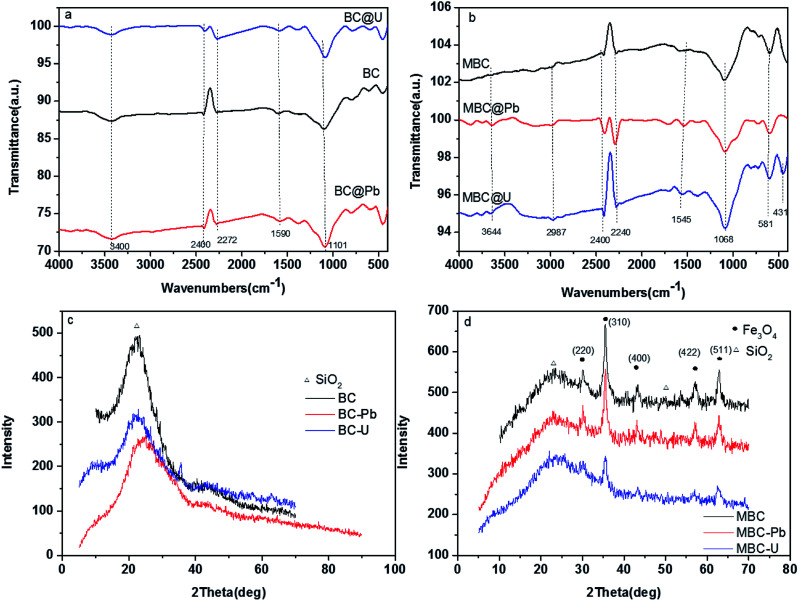
FTIR spectra and XRD patterns of BC before and after adsorption of Pb(ii) and U(vi) (a and c, respectively) and MBC before and after adsorption of Pb(ii) and U(vi) (b and d, respectively).

**Fig. 10:**
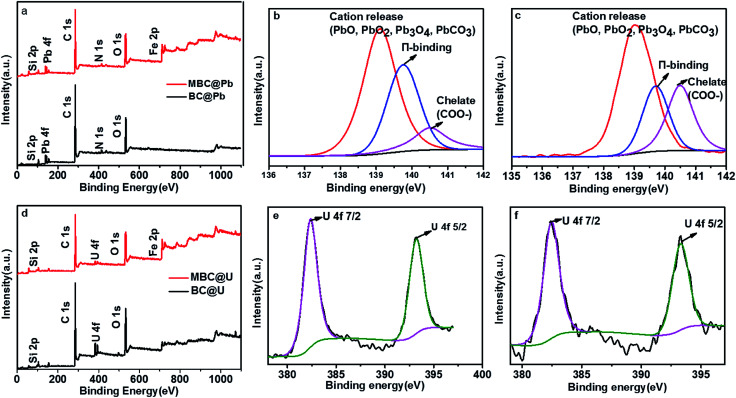
XPS wide scan (a and d) and high resolution Pb 4f (b and c) and U 4f (e and f) spectra of BC and MBC, respectively.

To investigate the adsorption mechanism of Pb(ii) and U(vi) on BC and MBC, FTIR, XRD, and XPS analyses were conducted before and after adsorption (Fig. 9 and 10). A band at 3420 cm^−1^ was observed and ascribed to the stretching vibration of O–H of both biochars.^49,55^ The peaks at *ca.* 2360, 1620, and 1068 cm^−1^ corresponded to the N–H bond, the stretching of the C=C bond, and the C–O–C vibration of BC and MBC, respectively.^55,56^ The main band of the FTIR spectra of BC and MBC was observed at 584 cm^−1^ and corresponded to the Fe–O bond.^54^ Numerous peaks appeared or disappeared after adsorption of Pb(ii) and U(vi) on BC and MBC. The change in the peak near 2240 cm^−1^ was produced by the vibration of the NO_3_^−^ ion in Pb(NO_3_)_2_ and U(NO_3_)_6_, thereby revealing the presence of these species formed by ion exchange after adsorption of Pb(ii) and U(vi). Compared to BC after adsorption, the signals corresponding to the surface function groups of MBC were slightly shifted. Thus, the signal at 1558–1566 cm^−1^ shifted to 1695–1701 cm^−1^ after Pb(ii) and U(vi) adsorption on MBC, thereby confirming the formation of metal chelation complexes on this sample, in line with the results reported by Samsuri *et al.*^36^ and Trakal *et al.*^23^

The XRD patterns further confirmed the different adsorption mechanisms (Fig. 9c and d). The diffraction peaks of BC slightly shifted after adsorption, thereby potentially suggesting a very stable crystal structure of this material. Additionally, the XRD results suggested that the adsorption process took place on the outer surface of BC mostly *via* physical interactions. With regard to MBC, numerous peaks disappeared and weakened after the adsorption of Pb(ii) and U(vi). These results might indicate that the adsorption of Pb(ii) and U(vi) on MBC was carried out *via* surface complexes formation and chemical precipitation process.^50^ Moreover, compared to Pb(ii), the intensity of the peaks corresponding to U(vi) on BC and MBC decreased in all cases, thereby indicating the prevalence of chemical adsorption processes. In contrast, Pb(ii) was mainly bonded to the biochar sample *via* physical adsorption processes.

Finally, XPS measurements were conducted to further analyze the adsorption mechanism (Fig. 10). As shown in Fig. 8, a Pb 4f band with three different binding energies in the range of 137–142 eV was observed for BC and MBC. The presence of a main peak at −139 eV (Pb–O) revealed that the predominant adsorption mechanisms were cation release and/or precipitation.^57^ The peak at 139.6 eV (π-binding) suggested another adsorption mechanism (*i.e.*, surface adsorption). The intensity of this π-binding signal was slightly higher for MBC as compared to BC, and this could be attributed to the presence of Fe oxides on the surface of MBC.^21^ The peak at 140.5 eV (chelate COO–) of Pb(ii) suggested the existence of an additional adsorption mechanism (*i.e.*, complexation with Pb(ii)).^23^ The higher intensity of this chelate COO– signal for MBC was indicative of an enhanced complexation adsorption after magnetic modification because of the presence of Fe oxides in the structure of MBC.^23^ As shown in Fig. 8, the U 4f band of BC and MBC (located between the C 1s and O 1s bands) involved two different binding energies (382 and 393 eV) corresponding to the U 4f_7/2_ and U 4f_5/2_ transitions, respectively. According to the FTIR results, the peaks of U 4f can be ascribed to the formation of C–O–UO_2_^+^ and COO–UO_2_^+^ species *via* surface complex formation and precipitation processes.

## Conclusion

The adsorption of Pb(ii) and U(vi) on biochar and the reusability of this material was greatly improved *via* magnetic modification, especially for Pb(ii) removal. The pH and the temperature significantly affected the adsorption behavior of Pb(ii) and U(vi) on BC and MBC. The adsorption experimental data were well fitted by Langmuir isotherm and pseudo-second-order kinetic models. The removal of U(vi) was significantly enhanced over MBC as compared to Pb(ii). Based on the FTIR, XRD, and XPS results, electrostatic interaction and surface complexation were separately suggested as the main mechanisms for the adsorption of Pb(ii) and U(vi) on biochars. Ion exchange and complexation were noticeably improved after impregnation of Fe oxide on BC. The resultant MBC material can be used as a potential adsorbent for Pb(ii) and U(vi). The recycling study confirmed the good regeneration properties and stability of MBC, making it suitable for the removal of Pb(ii) and U(vi) species in solution.

## Conflicts of interest

There are no conflicts to declare.

## Supplementary Material
